# Peak nasal inspiratory flow assessment of polyp size and response from SYNAPSE

**DOI:** 10.1016/j.jacig.2024.100327

**Published:** 2024-08-20

**Authors:** Amber U. Luong, Joshua M. Levy, Ludger Klimek, Richard J. Harvey, Jared Silver, Steven G. Smith, Abby Fuller, Robert Chan, Peter W. Hellings

**Affiliations:** a McGovern Medical School of the University of Texas Health Science Center, Houston, Tex; b Emory University School of Medicine, Atlanta, Ga; c Center for Rhinology and Allergology, Wiesbaden, Germany; d Macquarie Medical School, Sydney, Australia; e US Medical Affairs - Respiratory, GSK, Durham, NC; f Clinical Sciences, Respiratory, GSK, Durham, NC; g Clinical Statistics, Respiratory, GSK, GSK House, Brentford, United Kingdom; h Veramed Ltd, Twickenham, United Kingdom; i Clinical Sciences, Respiratory, GSK, GSK House, Brentford, United Kingdom; j Catholic University of Leuven, Leuven, Belgium

**Keywords:** Chronic rhinosinusitis with nasal polyps, nasal polyp score, patient-reported outcomes, peak nasal inspiratory flow, mepolizumab

## Abstract

**Background:**

In the phase III SYNAPSE study, mepolizumab plus standard of care reduced total endoscopic nasal polyp score (NPS) versus that with placebo in patients with chronic rhinosinusitis with nasal polyps.

**Objective:**

Our aim was to investigate relationships between NPS and (1) peak nasal inspiratory flow (PNIF) and (2) patient-reported outcomes.

**Methods:**

In this *post hoc* analysis, patients randomized 1:1 received mepolizumab, 100 mg, or placebo subcutaneously every 4 weeks (plus standard of care). Changes from baseline in PNIF (week 52), visual analog scale scores (overall symptoms, nasal obstruction, and loss of smell [weeks 49-52]), and total 22-Item Sino-Nasal Outcome Test score (week 52) were assessed in patients with or without improvements in NPS (improvement of ≥1 point) or without (improvement of <1 point or worsening).

**Results:**

Patients with improvements in NPS had greater improvements in PNIF (a median of 50 L per minute [interquartile range (IQR) = 10.5-87.5] with mepolizumab vs a median of 40 L per minute [IQR = 0-85.0] with placebo) than did those patients without improvements in NPS (a median of 0.0 L per minute [IQR = –10.0 to 45.0] with mepolizumab vs a median of 0.0 L per minute [IQR = –30.0 to 30.0] with placebo). Similar results were seen for the following: change from baseline in overall symptoms (a median of –5.8 [IQR = –8.1 to –3.80] with mepolizumab and a median of –4.1 [IQR = –7.0 to –1.2] with placebo with improvement in NPS vs a median of –1.3 [IQR = –6.3 to 0.0] with mepolizumab and a median of –0.1 [IQR = –3.4 to 0.0] with placebo without improvement in NPS); change in nasal obstruction (a median of –5.7 [IQR = –8.2 to –3.5] with mepolizumab and a median of –4.5 [IQR = –7.3 to –1.2] with placebo with improvement in NPS vs a median of –1.3 [IQR = –6.6 to 0.0] with mepolizumab and a median of 0.0 [IQR = –3.6 to 0.0] with placebo without improvement in NPS); change in loss of smell (a median of –2.8 [IQR = –7.9 to 0.0] with mepolizumab and a median of –0.7 [IQR = –4.0 to 0.0] with placebo with improvement in NPS vs a median of 0.0 [IQR = –2.4 to 0.0] with mepolizumab and a median of 0.0 [IQR = –0.3 to 0.0]) with placebo without improvement in NPS); and change in visual analog scale score and 22-Item Sino-Nasal Outcome Test total score (a median of –37.0 [IQR = –52.0 to –24.0] with mepolizumab and a median of –29.0 [IQR = –43.0 to –9.0] with placebo with improvement in NPS vs a median of –16.0 [IQR = –42.0 to 0.0] with mepolizumab and a median of 0.0 [IQR = –27.0 to 0.0] with placebo without improvement in NPS).

**Conclusion:**

Improvement in NPS was associated with improvements in PNIF and patient-reported outcomes irrespective of treatment. PNIF could be a useful noninvasive tool for monitoring nasal polyp size.

## Introduction

Chronic rhinosinusitis with nasal polyps (CRSwNP) is a chronic, inflammatory condition of the nose, paranasal sinuses, and upper airways.^1^ Common symptoms include nasal blockage, loss of smell, nasal discharge, and facial pain^1^ negatively impacting patients’ quality of life (QoL).^1^^,^^2^ The current standard of care (SoC) treatment includes intranasal corticosteroids, systemic corticosteroids, endoscopic sinonasal surgery, and biologics,^1^^,^^3^ with recent fine-tuning of the criteria for biologics.^2^

Mepolizumab is a humanized mAb inhibiting IL-5, which is a primary type 2 cytokine promoting eosinophil differentiation, activation, and survival.^4^^,^^5^ Mepolizumab is approved in the United States as an add-on maintenance treatment for patients with CRSwNP who are aged 18 years or older.^5^

In the phase III SYNAPSE study (GSK identifier 205687/ClinicalTrials.gov identifier NCT03085797) SoC with add-on mepolizumab significantly reduced nasal polyp (NP) size (measured by using total endoscopic NP score [NPS]) and sinonasal symptoms (measured by using the patient-reported outcomes [PROs] visual analog scale [VAS] score and 22-Item Sino-Nasal Outcome Test [SNOT-22] total score) versus placebo after 52 weeks in patients with severe treatment-refractory CRSwNP.^6^

A patient’s perception of his or her symptoms does not always correlate well with objective measures of NP size.^7^ PRO-based evaluation may be useful when nasal endoscopy is not available, especially when diagnostics are performed by non–ear, nose, and throat physicians. This *post hoc* analysis investigated the relationship between NPS and (1) change in peak nasal inspiratory flow (PNIF) and (2) PROs and PRO responder rates following treatment with mepolizumab or placebo.

The SYNAPSE trial design was described previously.^6^ Briefly, patients aged 18 years or older who had severe, recurrent, bilateral CRSwNP despite SoC treatment, significant nasal obstruction, and a history of 1 or more surgical procedures for NP removal (in the previous 10 years) were randomized (1:1) to receive mepolizumab, 100 mg, or placebo subcutaneously every 4 weeks (plus SoC treatment) for 52 weeks. Coprimary end points (change from baseline in NPS at week 52 and mean nasal obstruction VAS score at weeks 49-52) were described previously.^6^

This *post hoc* analysis assessed changes from baseline in PNIF (at week 52), sinonasal symptom VAS scores (overall symptoms, nasal obstruction, and loss of smell during weeks 49-52), and SNOT-22 total score (at week 52) among SYNAPSE patients with improvements from baseline (a ≥1-point decrease) in NPS versus among those without improvements (a <1-point decrease or an increase) in NPS at week 52. PNIF is a relatively low-cost, straightforward, and reliable measure to evaluate nasal patency in the clinic that correlates well with other objective assessments of nasal obstruction and patient-reported sinonasal symptoms in CRSwNP.^8^, ^9^, ^10^ Although some training is required for clinical staff to measure PNIF, this would not be expected to incur a significant training burden.^11^ PNIF was measured by using an IN-CHECK flow meter (Alliance Tech Medical, Granbury, Tex). After blowing their nose, patients inspired forcefully from residual volume to total lung capacity with their mouth closed. All measurements were made in the sitting position, ensuring a good seal around the face mask. The highest value of 3 consecutive (maximal) readings was recorded for each patient. Sinonasal symptom VAS scores, recorded daily with a 24-hour recall period, ranged from 0.0 to 10.0 (with 0 indicating total absence of symptom[s] and 10 indicating the worst thinkable severity).^6^ SNOT-22 total scores, recorded every 4 weeks with a 2-week recall period, ranged from 0 to 110 (from 0 to 5 per question, with higher scores indicating worse QoL), with a minimal clinically important difference of 8.9.^6^^,^^12^ NPS was defined as the sum of the right and left nostril scores (scale, 0-4 per nasal cavity; 0-8 total); higher scores indicated worse status.^6^ Proportions of PRO responders (patients with improvements in overall symptom VAS scores of 2.5 or more points, nasal obstruction/loss of smell of 3 or more points, and SNOT-22 score of 8.9 or more points) were also described for patients with or without improvements from baseline in NPS at week 52. Patients withdrawing from the study, missing week 52 data, or undergoing sinonasal surgery during the study were assigned their worst possible score immediately before withdrawal and were included in the “no NPS improvement” subgroup. Statistical analysis details were published previously.^6^

## Results and discussion

**Fig 1:**
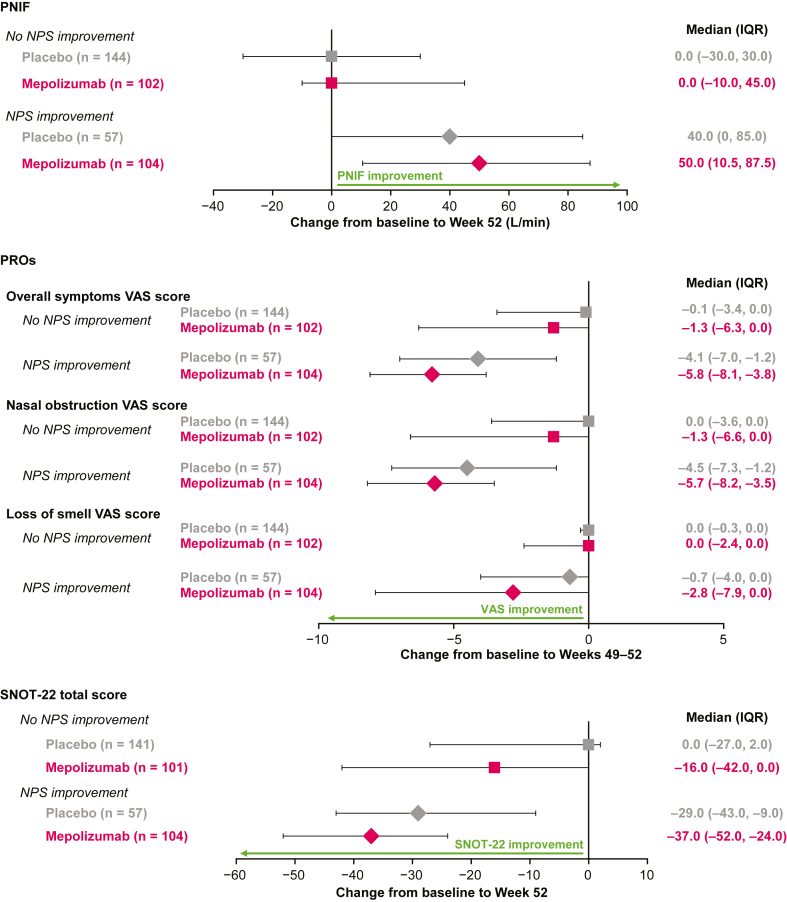
Change from baseline in PNIF, overall symptoms, nasal obstruction and loss of smell VAS score and SNOT-22 total score for patients with versus without at least a 1-point improvement in NPS by treatment arm. *IQR*, Interquartile range.

**Fig 2:**
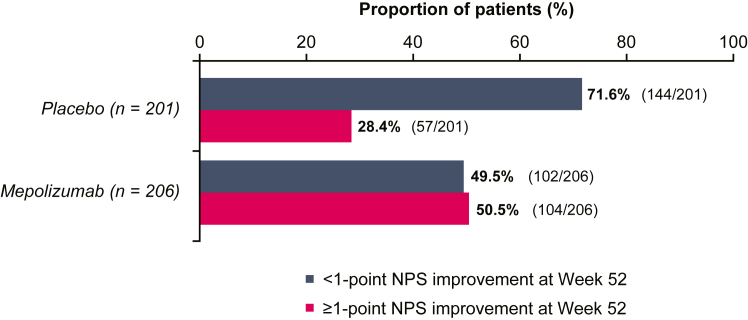
Proportion of patients who had at least a 1-point improvement or less than a 1-point improvement in NPS at week 52 by treatment arm. Patients who underwent sinus surgery, withdrew from the study, or who had missing data at week 52 were assigned their worst observed value immediately before the time point of their surgery, withdrawal, or missing data.

At baseline, patients in the mepolizumab and placebo groups had similar mean (SD) NPSs (5.4 [± 1.2] vs 5.6 [± 1.4], respectively). Sinonasal symptoms VAS scores and SNOT-22 scores at baseline were similar between treatment groups (Table I). Those patients with NPS improvements at week 52 had larger improvements in median PNIF at week 52 (50 L per minute with mepolizumab vs 40 L per minute with placebo) than did those who had no NPS improvements (0.0 L per minute vs 0.0 L per minute), regardless of treatment arm (Fig 1). A higher proportion of patients receiving mepolizumab than placebo achieved improvements in NPS of at least 1 point at week 52 (50% vs 28%, respectively) (Fig 2). Those patients with improvements in NPS had numerically larger improvements in median change from baseline in overall symptoms VAS score (–5.8 with mepolizumab vs –4.1 with placebo) than did the patients without NPS improvements (–1.3; –0.1 respectively) at weeks 49 to 52 (Fig 1); patients receiving mepolizumab had numerically larger improvements than those receiving placebo did, regardless of NPS improvement. Similar results were observed for nasal obstruction VAS, whereas slight improvements in loss of smell VAS were observed in patients with improvements in NPS (–2.8 with mepolizumab vs –0.7 with placebo) (Fig 1). Patients who had NPS improvements reported larger change from baseline in SNOT-22 total score at week 52 (–37.0 with mepolizumab vs –29.0 with placebo) than did patients with no NPS improvement (–16.0 and 0.0, respectively). Overall, patients treated with mepolizumab reported larger improvements in SNOT-22 total scores than did those who received placebo, regardless of improvement in NPS (Fig 1).

**Table I:** Mean baseline VAS scores, SNOT-22 scores, and PNIF per NPS improvement and treatment arm

Outcome measure	Placebo (n = 201)		Mepolizumab (n = 206)	
	<1-point NPS improvement	≥1-point NPS improvement	<1-point NPS improvement	≥1-point NPS improvement
VAS scores (scale of 1-10), mean (SD)				
Overall symptoms VAS	(n = 144) 9.1 (0.7)	(n = 57) 9.1 (0.7)	(n = 102) 9.0 (0.8)	(n = 104) 9.0 (0.8)
Nasal obstruction VAS	(n = 144) 9.0 (0.9)	(n = 57) 9.0 (0.7)	(n = 102) 8.9 (0.8)	(n = 104) 8.9 (0.8)
Loss of smell VAS	(n = 144) 9.7 (0.5)	(n = 57) 9.6 (0.7)	(n = 102) 9.7 (0.6)	(n = 104) 9.6 (1.0)
SNOT-22 total score (scale of 1-110), mean (SD)	(n = 141) 65.7 (19.3)	(n = 57) 61.3 (18.2)	(n = 101) 65.6 (17.9)	(n = 104) 61.8 (17.2)
PNIF (L per min), mean (SD)	(n = 144) 103.0 (78.1)	(n = 57) 97.9 (43.6)	(n = 102) 103.8 (61.4)	(n = 104) 99.3 (55.5)

**Fig 3:**
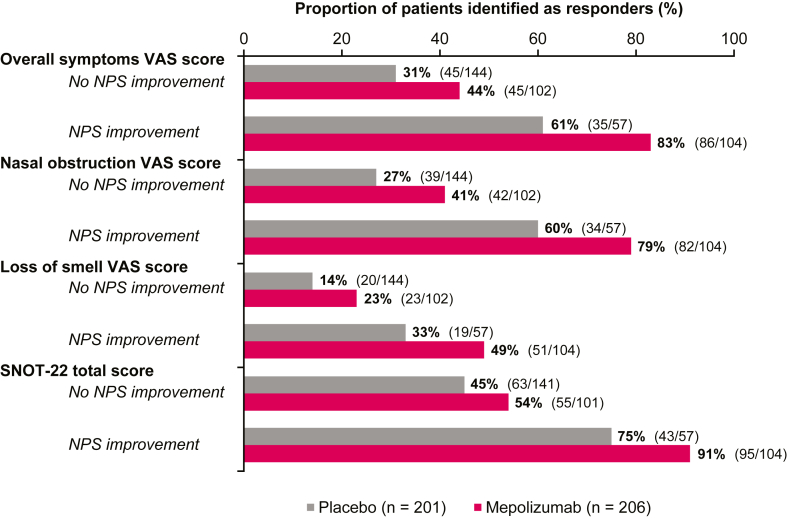
Proportions of patients identified as PRO responders for those patients with or without at least a 1-point improvement in NPS from baseline to week 52 by treatment arm.

A larger proportion of patients with improvements in NPS at week 52 were identified as overall sinonasal symptom VAS score responders than were patients without NPS improvements (Fig 3). Response rates were higher for patients treated with mepolizumab (83% vs 61% in the case of responders with NPS improvements as opposed to 44% vs 31% in the case of with no NPS improvements, respectively). Most patients receiving mepolizumab who had NPS improvements were identified as responders in terms of overall symptoms VAS, nasal obstruction VAS, and SNOT-22 total score (83%, 79%, and 91%, respectively) (Fig 3).

This *post hoc* analysis of SYNAPSE data suggests that for patients with severe CRSwNP, an improvement in NPS of at least 1 point from baseline to week 52 is associated with increased PNIF and larger improvements in sinonasal symptoms, irrespective of treatment. This in turn suggests that PNIF could be a useful noninvasive tool for monitoring NP size when endoscopy is unavailable or delayed. For example, it may be useful when assessing patients after initial consultation or for monitoring patients over time during follow-up visits, particularly as it is simple to use, is reliable, and has added clinical utility when used in routine practice in conjunction with validated PRO measures.^10^ The finding that patients with NPS improvements had improvements in PNIF irrespective of treatment arm may suggest that PNIF reflects NP size directly rather than simply also reflecting improvement in response to biologic therapy (eg, through a reduction in sinonasal inflammation, which is also associated with improved PNIF).^13^ The change from baseline in PNIF in these patients was still 10 L per minute greater on average with mepolizumab than with placebo at week 52, which could suggest a biologic treatment effect or be reflective of numerically greater NPS improvements for patients treated with mepolizumab than with placebo within the NPS improvement group.

Furthermore, NPS improvements were generally concordant with robust PROs linked to nasal obstruction/congestion irrespective of treatment, thus highlighting the potential of NPS as a tool to evaluate disease burden for patients with CRSwNP. Although formal statistical tests were not performed, PRO improvements and proportions of PRO responders were also numerically higher for patients treated with mepolizumab than with placebo, independent of NPS improvements.

PROs demonstrated numeric improvement trends even in the absence of NPS response; this trend was also evident in patients receiving placebo. These results may reflect the fact that PROs and objective measures (such as NPS) quantify different aspects of the patient experience in CRSwNP, and that patient-reported perceptions of symptom burden and QoL may not always be directly influenced by NP size.^7^ Additionally, SNOT-22 assesses outcomes beyond solely nasal/sinus symptoms, such as sleep quality and extranasal symptoms.^14^ A holistic approach to CRSwNP management, in which multiple different measures are assessed, is important to understand the true patient benefits associated with treatment.

Although these data provide insights into associations between NPS, nasal airflow, patient-reported sinonasal symptoms, and QoL, caution is needed when interpreting the findings owing to the study’s *post hoc* and exploratory nature. Although a numeric correlation between PNIF and NPS is shown, a statistical test for correlation between NPS and other clinical parameters evaluated was deemed inappropriate, as the patients enrolled in SYNAPSE had severe NPs, resulting in a limited NPS range at baseline and a change from baseline that was rarely extreme in either direction. Furthermore, PNIF was not a powered end point of SYNAPSE.

This analysis shows that NPS improvements correspond well with PNIF improvements, suggesting that PNIF could be a useful noninvasive tool for monitoring NP size when endoscopy is unavailable. Although patients with NPS improvements also reported improved PROs, NPS was less concordant with PROs than PNIF was. Even without an NPS response, PROs consistently demonstrated trends toward improvement. Together, these data highlight the value of measuring several different clinical outcomes to improve assessment of disease burden and treatment response in patients with CRSwNP.

**Clinical implications**

Improved NPS was associated with improved PNIF and patient-reported outcomes in the SYNAPSE study. PNIF could be a useful noninvasive tool for monitoring NP size.

## Disclosure statement

This study and the *post hoc* analyses were funded by GSK (GSK identifier 205687/ClinicalTrials.gov NCT03085797). Medical writing and editorial support (in the form of writing assistance, including development of the initial draft based on author direction, assembly of tables and figures, collation and incorporation of authors’ comments on the drafts, grammatical editing, and referencing) was funded by GSK. The sponsor did not place any restrictions on access to the data or on the statements made in the article.

Disclosure of potential conflict of interest: A. U. Luong serves as a consultant for Aerin Medical, GSK, Lyra Therapeutics, Medtronic, NeurENT Medical, Sanofi, and Stryker and also serves on scientific advisory boards for Maxwell Biosciences and SoundHealth. J. M. Levy reports serving as a consultant for 10.13039/100004325AstraZeneca, GSK, 10.13039/100019966Honeywell International, and Regeneron; receiving grants from Honeywell International, Sanofi/Regeneron, Genentech, and the 10.13039/100000002National Institutes of Health (grants 1R03TR004022-01 and HL-143541-02S2). L. Klimek reports grants and personal fees from 10.13039/100009946Allergopharma, Novartis, Bionorica, GSK, and Lofarma; personal fees from MEDA and Boehringer Ingelheim; and grants from AstraZeneca, Biomay, HAL, LETI Pharma, Roxall, Sanofi, and Bencard outside the submitted work. R. J. Harvey reports serving as a consultant/advisory board member with Medtronic, Novartis, Neilmed, Sanofi, GSK, and Viatris Pharmaceuticals; receiving research grant funding from GSK and Stallergenes; and having served on the speakers bureaus for Stallergenes, Sanofi, GSK, AstraZeneca, Viatris Pharmaceuticals, and Seqirus. A. Fuller is a contract resource for GSK. P. W. Hellings has received research grants and/or consultancy or lecture fees from Sanofi, Regeneron, GSK, Novartis, Viatris, and AstraZeneca. J. Silver, S. G. Smith, and R. Chan are employees of GSK and own stock/shares.
